# Homo- and Dikaryons of the Arbuscular Mycorrhizal Fungus *Rhizophagus irregularis* Differ in Life History Strategy

**DOI:** 10.3389/fpls.2021.715377

**Published:** 2021-08-05

**Authors:** Edward Umberto Serghi, Vasilis Kokkoris, Calvin Cornell, Jeremy Dettman, Franck Stefani, Nicolas Corradi

**Affiliations:** ^1^Department of Biology, University of Ottawa, Ottawa, ON, Canada; ^2^Agriculture and Agri-Food Canada, Ottawa Research and Development Centre, Ottawa, ON, Canada

**Keywords:** mycorrhiza, fungi, functional traits, AMF dikaryosis, life history strategies, hyphal growth, hyphal network

## Abstract

Arbuscular mycorrhizal fungi (AMF) are obligate plant symbionts that have the potential to improve crop yield. These multinucleate organisms are either “homokaryotic” or “dikaryotic”. In AMF dikaryons, thousands of nuclei originating from two parental strains coexist in the same cytoplasm. In other fungi, homokaryotic and dikaryotic strains show distinct life history traits (LHTs), such as variation in growth rates and fitness. However, how such traits compare between dikaryons and homokaryons of AMF is unknown. To address this, we measured 20 LHT of four dikaryons and five homokaryons of the model fungus *Rhizophagus irregularis* across root organ cultures of three host plants (carrot, chicory, and tobacco). Our analyses show that dikaryons have clearly distinct life history strategies (LHSs) compared to homokaryons. In particular, spores of homokaryons germinate faster and to a higher proportion than dikaryons, whereas dikaryons grow significantly faster and create a more complex hyphal network irrespective of host plant species. Our study links AMF nuclear status with key LHT with possible implications for mycorrhizal symbiotic functioning.

## Introduction

Arbuscular mycorrhizal fungi (AMF) are a group of ubiquitous soil fungi that form symbioses with the roots of most land plants (Kivlin et al., 2011; Davison et al., 2015; Brundrett and Tedersoo, 2018). In presence of AMF, plants usually grow better and produce higher yields (Hoeksema et al., 2010), particularly under stressful environmental conditions (Evelin et al., 2009). Due to the benefits that AMF provide to their host plants, many industries produce AMF inocula to enhance plant growth in agriculture, forestry, horticulture, phytoremediation, and nurseries (Gianinazzi and Vosatka, 2004).

The AMF harbor thousands of nuclei within a common cytoplasm (Kokkoris et al., 2020a), and it has been demonstrated that strains of the model species *Rhizophagus irregularis* are either “homokaryotic” (AMF homokaryons)—or heterokaryotic (AMF dikaryons) (Ropars et al., 2016; Corradi and Brachmann, 2017; Chen et al., 2018). In homokaryons, all coexisting nuclei are genetically similar harboring one putative mating-type (MAT)-locus, whereas in dikaryons, two genetically unique nuclear populations, each harboring a unique MAT-locus, co-exist at stable or variable ratios depending on the plant host (Kokkoris et al., 2021). A MAT-locus is a region that governs sexual identity in fungal strains, and the MAT-locus of *R. irregularis* resembles the MAT-locus of basidiomycetes (Casselton, 2008).

In other fungal phyla (e.g., Ascomycota and Basidiomycota), dikaryosis is defined by the presence of two parental nuclei per cell, and this feature can sometimes provide a functional advantage to dikaryotic strains. For example, dikaryotic fungal strains can be superior in terms of fitness and function (i.e., unique proteins expression, increased enzymatic activity, improved symbiotic ability, etc.) compared to the homokaryotic parents (Wagner et al., 1988; Clark and Anderson, 2004; Gehrmann et al., 2018). Furthermore, changes in the ratios of co-existing nuclei can result in distinct fungal phenotypes and confer adaptability in changing environments (Jinks, 1952).

In AMF, the ratio of co-existing parental nuclei is also actively regulated in response to the host plant identity (Kokkoris et al., 2021), yet the phenotypic consequences of this regulation are currently unknown. More generally, how AMF homokaryons and dikaryons compare in terms of growth and symbiotic potential is unclear. Here, we hypothesize that the presence of higher genetic diversity and host-dependent nuclear dynamics can provide dikaryons an adaptive advantage in response to changing plant partners. To test this, we performed two separate experiments that examined the life history traits (LHTs) of homokaryotic and dikaryotic strains of *R. irregularis*.

In the first experiment, we grew six homokaryotic and four dikaryotic strains (out of five available in culture collections) in the absence of a host to evaluate variation in LHTs during the asymbiotic growth stage. In the second experiment, we grew the same strains (except the strain Cuba8, which failed to germinate during the first experiment) with three different host plant species to evaluate the LHT differences between dikaryotic and homokaryotic strains during the symbiotic growth and their response to different host plant species. Overall, 20 phenotypic traits, including spore and hyphal morphological traits and fitness-related traits, such as germination ability, speed of hyphal growth, and complexity of hyphal network, were measured using photomicrography and image analysis.

## Materials and Methods

### Arbuscular Mycorrhizal Fungi

We used 10 strains of *R. irregularis* (Schenck and Smith). Specifically six homokaryotic strains: **Cuba8** (DAOM 984909), **330** (DAOM229455), **66** (DAOM240720), **197198** (DAOM197198), **101** (DAOM240448), and **98** (DAOM240446) and four dikaryotic strains: **A4** (DAOM664343), **A5** (DAOM664344), **SL1** (DAOM240409), and **G1** (DAOM970895), which were recently analyzed for nuclear dynamics (Kokkoris et al., 2021). The strains were obtained from the Canadian Collection of Arbuscular Mycorrhizal Fungi (CCAMF), previously known as Glomeromycota *In vitro* Collection—(GINCO) and from the dikaryotic AMF collection—Corradi Lab at University of Ottawa. All the strains were grown and maintained *in vitro* with Ri T-DNA-transformed roots of *Daucus carota* cv. P68, growing in M-medium (Bécard and Fortin, 1988) solidified with 3% Phytagel (Sigma-Aldrich, St. Louis, MO, United States) in dual compartment Petri dishes. These strains were selected based on their MAT-loci in order to account for the MAT variation encountered in the dikaryons and their phylogenetic differences—i.e., they cluster in three distinct clades as reported in Ropars et al. (2016); Savary et al. (2018), and Kokkoris et al. (2021) (Supplementary Table 1).

### AMF Spore Extraction

Spores were extracted from the monoxenic *in vitro* cultures after dissolving pieces of the spore-containing medium, using sodium citrate buffer (pH 6.0; at 30°C) (Doner et al., 1991). Spores were then stored at 4°C for 20 days to break the spore dormancy and reduce spore mortality (Juge et al., 2002) before initiating the experiments.

**Experiment 1**: Asymbiotic trait variation between homokaryotic and dikaryotic strains of *R. irregularis*.

### Experimental Design

We followed a similar approach as used in Kokkoris et al. (2019a). Briefly, we chose 40 healthy-looking spores per strain (*n* = 400), with intact spore walls and no discoloration. Spores were imbedded individually in the center of 60 mm × 15 mm Petri dishes filled with M-medium (Bécard and Fortin, 1988) solidified with 3% Phytagel (Sigma-Aldrich, St. Louis, MO, United States). The Petri dishes were sealed with parafilm and incubated in a growth chamber (Precision, Thermo Fisher Scientific, Waltham, MA, United States) at 26°C in the dark.

### Image Acquisition and Trait Quantification

We examined multiple LHTs during the AMF asymbiotic growth stage (Table 1). Initially, we used a dissecting microscope and the software Infinity Capture (Lumenera^®^ software, Ottawa, ON, Canada) to take pictures of all plated spores. The *spore area* and the *spore diameter* were evaluated in each image by using the oval selection and brush selection tools, and the straight-line tool in FIJI—ImageJ v. 1.53c (Schindelin et al., 2012).

**TABLE 1:** Life history traits (LHTs) measured in two experiments along with the abbreviation and the description of each trait.

Trait	Code	Description
**Asymbiotic**		
Spore area	SA	The surface area of each spore
Spore diameter	SD	The length from one side of the spore to the opposite. For non-circular spore we determined the largest diameter
Germination tubes	GT	The number of germ tubes growing from either the spore wall or from the subtending hypha
Hyphal tips	HT	The total number of hyphal tips 30 days post-germination, not including the subtending hypha
Hyphal reach (exploration ability)	HR	The distance from the center of the spore to the furthest hyphal tip 30 days post-germination
Hyphal diameter	HD	The mean diameter of a hypha
Time to germination	TG	The number of days to germination tube emergence
Total hyphal length	THL	The total hyphal length 30 days post-germination
Branching intensity = Hyphal length/Hyphal tips	BI	The branching intensity calculated by the total hyphal length divided by the number of hyphal tips.
Total number of septae	NS	The total number of septae 30 days post-germination
Septae formation per hyphal unit	S	The total number of septae divided by the total hyphal length
Hyphal growth rate	HGR	Total hyphal length divided by the number of days it took for a spore to stop growing/end of experiment (um per day)
Spore percent germination	SG	The number of spores that germinated successfully divided by the total number of plated spores for each particular strain
**Symbiotic**		
Days to extraradical mycelium (ERM) emergence	DERM	The time required for the extraradical mycelium to emerge
Time to sporulation	TSPOR	The time it takes the strain to form spores (in any compartment)
Hyphal exploration speed	HSPEED	The average distance a strain can explore per day
Branching factor	BF	Complexity of hyphal network in the hyphal compartment defined based on the hyphal branching intensity
Total hyphal length	HL	Length of the hyphae in the hyphal compartment a month after crossing the bridge to this compartment
Number of spores	SPORES	Total number of spores in the hyphal compartment a month after crossing the bridge to this compartment
Hyphal network density	ND	The amount of medium as determined by counting the total number of hyphal pixels in the defined region vs. background pixels

Each Petri dish was examined every 2 days for germination using a dissecting microscope ZEISS Stemi 305 (Toronto, ON, Canada). With germination as a starting point, each spore was left to grow for 30 additional days, and the number of germination tubes, hyphal reach (HR; μm), total hyphal growth (μm), number of septae, and number of tips were quantified at the end of this period using microphotography and image processing. In detail, the number of germination tubes and number of septae were manually counted using a Nikon Eclipse 800 microscope/Nikon digital sight microscopy camera (Minato City, Tokyo, Japan) (DS-Ri2) and the software NIS-Elements BR version 4.60. We used the “add trace” tool (Neuron J Plugin in FIJI—ImageJ v. 1.53c) (Schindelin et al., 2012) on the acquired images, which fits a line to the hyphae semiautomatically, to measure the total hyphal length. We used the “add line tool” in FIJI—ImageJ v. 1.53c to measure the HR. Images were stitched together in Adobe Photoshop version: 22.1.1 (San Jose, CA, United States) when necessary to quantify the total hyphal length and the HR. To standardize the number of septae between the strains, we divided the total number of septae by the total hyphal length to calculate the number of septae per hyphal length. Finally, the branching intensity was calculated by dividing the number of hyphal tips by the total hyphal length.

**Experiment 2**: Symbiotic trait variation between homokaryotic and dikaryotic strains of *R. irregularis*.

### Root Organ Cultures

To eliminate external confounding effects (e.g., microbial interactions, local abiotic variation, etc.) commonly affecting field and in-planta studies, and thus to maximize detection probability of genetic effects, all AMF strains investigated here were propagated under stable growth conditions with receiver operating characteristics (ROCs). Despite its artificial nature (Kokkoris and Hart, 2019a), AMF culturing on ROC is the best available system to obtain unbiased information on LHTs of AMF—since hyphal growth and network complexities are otherwise hidden and concealed within soil environments and cannot be easily observed and measured (Koch et al., 2004; Azcon-Aguilar et al., 2017). Furthermore, the sterility of the system limits unwanted interactions from exogenous factors, such as bacteria in sandwich systems in Petri plates filled with sterile grit (e.g., see Pepe et al., 2017; Sbrana et al., 2020).

We used ROCs of three plant species that have been shown in the past to trigger responses in the nucleotype ratios of the dikaryotic strains (Kokkoris et al., 2021). The species used were *Daucus carota* (Carrot) cv. P68, *Cichorium intybus* (Chicory), and *Nicotiana benthamiana* (Nicotiana) cv. benthamiana.

### Experimental Design

Each experimental unit consisted of a dual-compartment Petri dish with the “root compartment” containing an individual ROC and the AMF spores, and the “fungal compartment” purposed to hold only the growing fungal tissue (hyphae and spores). The two compartments were united with a sterile filter paper bridge to facilitate hyphal crossing to the hyphal compartment. Seven replicates were plated for each of the 27 plant host × AMF strain combinations (*n* = 189, i.e., three ROCs and nine *R. irregularis* strains).

### Transformed Root Culture Setup

Both split-plate compartments were filled with M-medium (Bécard and Fortin, 1988) solidified with 3% Phytagel (Sigma-Aldrich, St. Louis, MO, United States). The root compartment was supplemented with sucrose (10 g/L) to allow for root growth (Bécard and Fortin, 1988), whereas the fungal compartment was left sucrose free to promote sporulation. We standardized the propagule density to 10 viable spores per strain based on the spore germination ability of each strain as evaluated from our first experiment (see result section for Experiment 1 and Supplementary Table 1). Although the germination conditions for Experiment 2 were not the same as in our first experiment (presence of ROC host), and while the presence of a host has been demonstrated to stimulate spore germination and to promote hyphal growth (Bécard and Piché, 1989; Bécard et al., 1992), the low number of propagules per strain (10 viable propagules) used in our experiment aimed to dilute any potential host effect on germination. In accordance with the first experiment, spores with intact spore walls and no discoloration were chosen. The initial ROC fragments were standardized based on length and age, and we specifically used young root tips of 3 cm in length for all experimental units to reduce any interactive effects (Powell, 1976; Bécard and Fortin, 1988). Spores and roots were plated at the same time and positioned at the far end of the root compartment, furthest away from the paper bridge between the compartments. Similar to our first experiment, the plates were incubated in a growth chamber (Precision, Thermo Fisher Scientific) at 26°C in the dark. The plant roots were periodically redirected to prevent them from growing into the fungal compartment. Contaminated plates or plates where the AMF failed to germinate or colonize the host were discarded.

### Image Acquisition and Trait Measurements

We examined multiple LHTs during the symbiotic growth stage (Table 1). The number of days until emergence of the extraradical mycelium (ERM) was recorded manually after daily observation of the Petri plates using a dissecting microscope (ZEISS Stemi 305). *Time to sporulation* (in days) was also assessed through daily culture observation.

To assess the *hyphal exploration speed*, semicircles (1 cm intervals) were drawn on the hyphal compartment side of each Petri plate lid. Starting from the time point, the hyphae crossed the filter paper bridge to the fungal compartment, we recorded the time (in days) required for the hyphae to reach each of the three semicircles. In the end, we calculated the average time it took for the hyphae to reach as far as 1 cm (see Supplementary Figure 1 for method details).

The *total hyphal length* was quantified 30 days after the hyphae entered the hyphal compartment. We used a microscope Zeiss AxioZoom.V16 (Toronto, ON, Canada) and the function “panorama” in the ZEN 2.3 software (Carl Zeiss MicroImaging, Göttingen, Germany) to acquire complete stitched images of the entire fungal compartment. The hyphal length in the entire fungal compartment was traced semi-automatically using the “add trace” tool (Neuron J Plugin in FIJI—ImageJ v. 1.53c) (Schindelin et al., 2012).

The total *number of spores* was quantified manually in the entire hyphal compartment using the function “cell counter” in FIJI—ImageJ v. 1.53c (Schindelin et al., 2012). The spores that were produced from the strains growing with tobacco host plant were quantified in the root compartment since none of the strains produced spores in the fungal compartment.

To quantify branching and hyphal network density, we used the software RhizoVision Analyzer (version 2.0.0 beta© 2018–2020 Noble Research Institute, LLC, Ardmore, OK, United States) (Seethepalli et al., 2021). For the fungal compartment, we defined three regions of interest (1.5 cm × 1.5 cm)—on the left, center, and right side of the plate—with the parameters “invert image” and “filter noisy components of the image” selected, with “Root type = disconnected root” and by adjusting the imaging threshold level manually to provide the most accurate read. This analysis provides the hyphal branching value and hyphal network area. The “Network Area” of the image is determined by counting the total number of hyphal pixels in the defined region (higher value = denser hyphal network).

**FIGURE 1:**
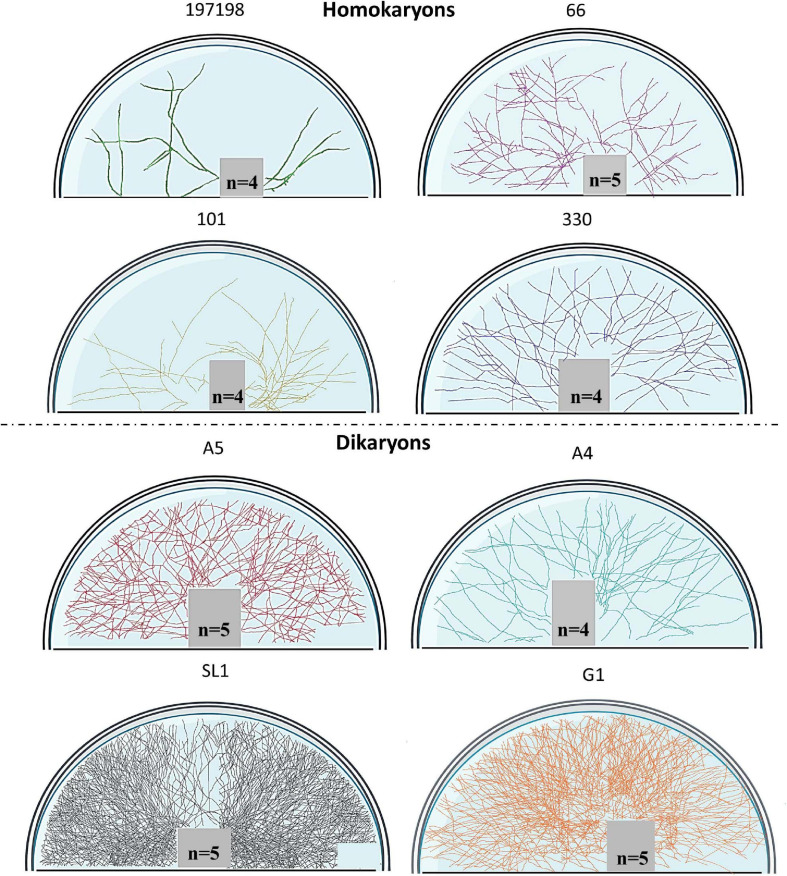
Symbiotic hyphal growth of four homokaryotic and four dikaryotic strains in *Cichorium intybus* root organ cultures as a host. The images demonstrate the actual hyphal growth per strain 30 days after the hyphae crossed to the hyphal compartment of the Petri dish. The bridge and the lower right part of the Petri dish appear empty since hyphal growth could not be accessed as it was concealed by either the bridge or by the blurred part of the Petri dish (manufacturers feature). To correct for size variation of the filter bridge between plates, an area was removed from each image prior to determination of the hyphal traits. Although multiple plate replicates were analyzed per strain (*n* number visible in each plate), we chose to demonstrate one representative plate per strain.

**FIGURE 2:**
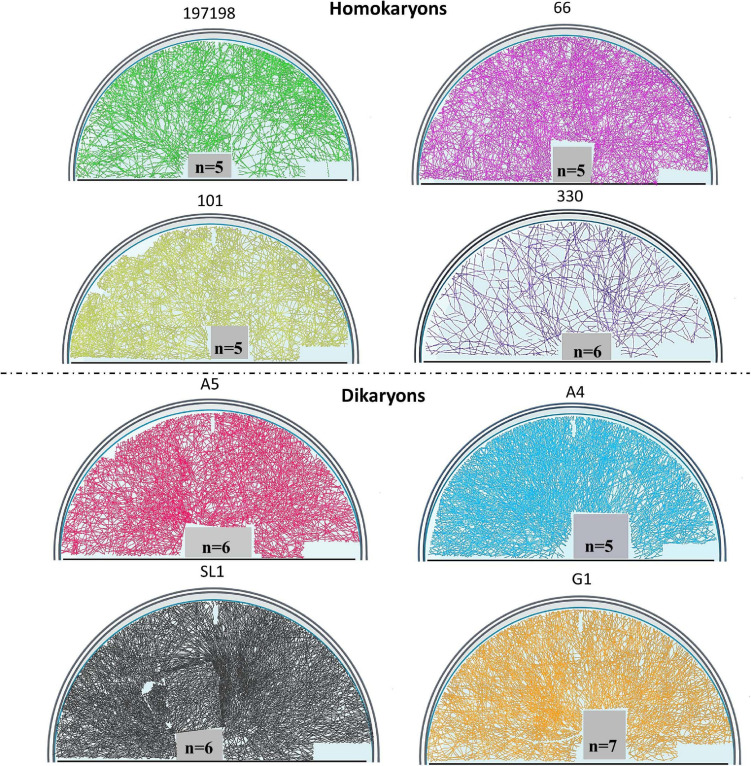
Symbiotic hyphal growth of four homokaryotic and four dikaryotic strains in *Daucus carota* root organ culture as a host. The images demonstrate the actual hyphal growth per strain 30 days after the hyphae crossed to the hyphal compartment of the Petri dish. The bridge and the lower right part of the Petri dish appear empty since hyphal growth could not be accessed as it was concealed by either the bridge or by the blurred part of the Petri dish (manufacturers feature). To correct for size variation of the filter bridge between plates, an area was removed from each image prior to determination of the hyphal traits. Although multiple plate replicates were analyzed per strain (*n* number visible in each plate), we chose to demonstrate one representative plate per strain.

To visualize entire mycelia, partial plate images were stitched together after tracing all hyphae in FIJI (NeuronJ) in the fungal compartment. We used Adobe Photoshop version 22.1.1. to extract the hyphal pattern and then we used BioRender (Toronto, ON, Canada) to create figures that demonstrate the actual hyphal growth patterns (Figures 1, 2 and also see Supplementary Figure 2 for the method details and Supplementary Figure 3 for additional original full plate images). We did not create such figures for tobacco since almost no hyphae propagated into the fungal compartment (none for the homokaryons and some hyphae for the dikaryons SL1 and A5).

### Statistical Analyses

#### Experiment 1

To explore whether dikaryons differ from homokaryons in terms of LHTs, we used a permutational multivariate analysis of variance (PERMANOVA) (Anderson, 2017). PERMANOVA was performed in R studio (version 1.3.1093© 2009–2020 RStudio, PBC, Boston, United States) using the R package: “Vegan version 2.5-3,” by running the function Adonis (Oksanen, 2015). PERMANOVA was performed with nuclear status (Homokaryosis vs. Dikaryosis) as fixed factor and strain identity as a random factor and by using the Euclidian distance metric as dissimilarity index and 999 permutations. Data were first standardized using the “max” method and the “decostand” command in R (package Vegan version 2.5-3) prior to the PERMANOVA. The results were visualized with a non-metric multidimensional scaling (NMDS) plot using the package ggplot2 (Wickham, 2010) in R studio. For this analysis, we excluded the traits “number of tips” and “number of septa” since they are included in the standardized measurement (septa per hyphal unit and branching intensity).

To examine individual traits on differences among dikaryons and homokaryons, we used the non-parametric Kruskal–Wallis test with nuclear status (Dikaryosis vs. Homokaryosis) as a fixed factor. The strain Cuba8 (DAOM 984909) was excluded from the analysis since the spores of this strain consistently failed to germinate under the conditions of our study, possibly due to non-viable stock material. Overall, four dikaryons (A4, A5, SL1, and G1) and five homokaryons (330, 66, 197198, 101, and 98) were compared.

#### Experiment 2

We used a similar approach as in our first experiment to explore whether the dikaryons differ from the homokaryons in the measured LHTs during the symbiotic growth stage. PERMANOVA was performed with nuclear status (Homokaryosis vs. Dikaryosis) and host plant identity (chicory, carrot, and tobacco) as fixed factors, also examining the interaction between the two and accounting for the nestedness of our experimental design (Nuclear organization/Host plant), and the strain identity was considered as a random factor. The parameters of the PERMANOVA were “Euclidian distance” as dissimilarity index and 999 permutations. Data were standardized using the “max” method and the “decostand” command in R (package Vegan version 2.5-3) prior to the PERMANOVA. The results were visualized by NMDS as for Experiment 1.

To examine traits individually when the residuals were normally distributed, we used linear mixed effect models (lmer command—R package lme4 version 1.1-23; Bates et al., 2015) with nuclear status and host plant identity as fixed factors (also examining the interaction) also accounting for the nestedness of the experimental design (Nuclear organization/Host plant), and strain identity as random factor. Generalized Linear Mixed models (glm—R package lme4 version 1.1-23; Bates et al., 2015) were used when the residuals of the model were not normally distributed (even after transformation). Strain 98 (DAOM240446) failed to colonize chicory and nicotiana ROCs and colonized only one plate with carrot ROC and was therefore excluded from the analysis. Overall, there were four dikaryons (A4, A5, SL1, and G1) compared to four homokaryons (330, 66, 197198, and 101).

## Results

**Experiment 1**: *Asymbiotic variation between homokaryotic and dikaryotic strains of R. irregularis.*

**FIGURE 3:**
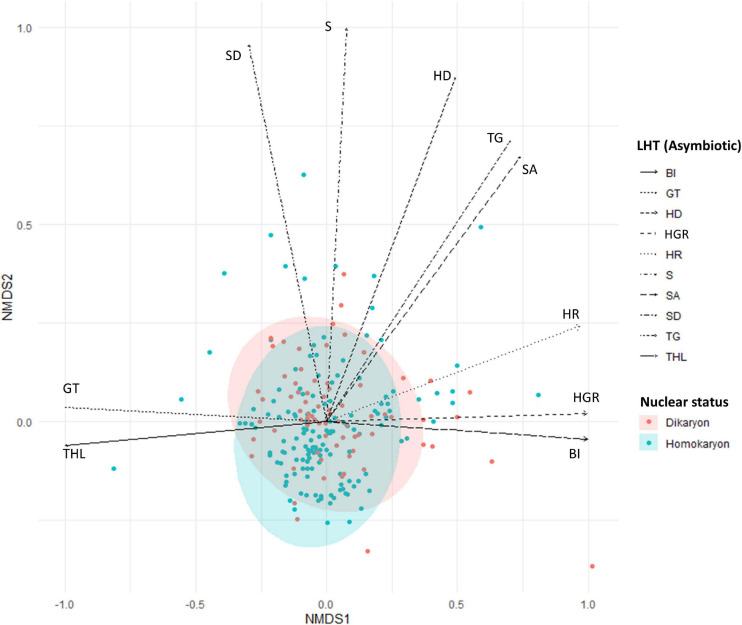
Non-metric multidimensional scaling (NMDS) plot of homokaryotic (blue dots) and dikaryotic (red dots) strains based on the measured life history traits during the asymbiotic growth stage. Stress value = 0.1. The direction of the traits was obtained after fitting the measured traits in the ordination space. SD, spore diameter; SA, spore area; GT, number of germ tubes; HR, hyphal reach; HD, hyphal diameter; TG, time to germination; THL, total hyphal length; BI, branching intensity; S, septae per hyphal unit; HGR, hyphal growth rate. Blue and red ellipse are 95% CI.

Overall, we observed a significant difference between homokaryons and dikaryons (Pseudo-*F* = 6.04, *p* = 0.001) when considering all 13 asymbiotic traits together (Figure 3 and Supplementary Figure 4).

**FIGURE 4:**
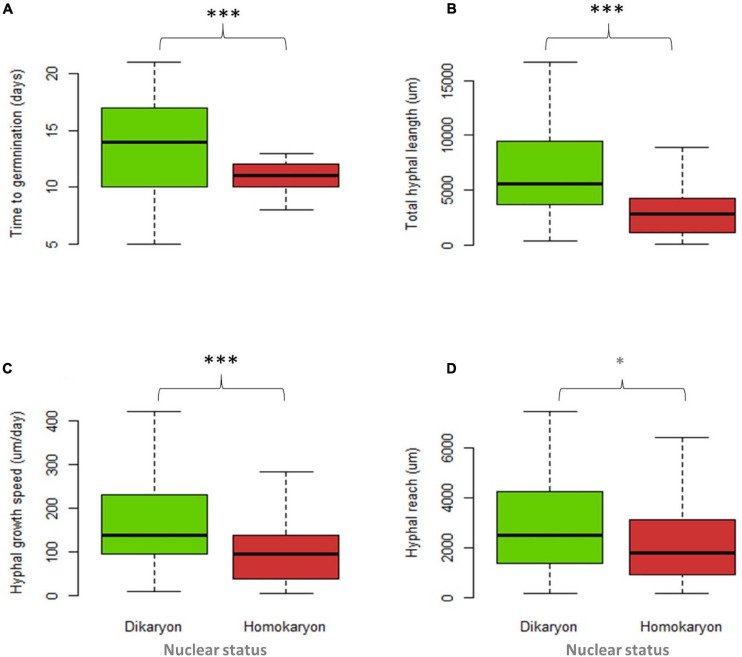
Trait variation between four dikaryotic (green) and five homokaryotic (red) strains of *Rhizophagus irregularis* during the asymbiotic growth stage. **(A)** Time to germination, **(B)** total hyphal length, **(C)** hyphal growth speed, and **(D)** hyphal reach. Statistical significance was evaluated using the non-parametric Kruskal–Wallis test. The asterisks indicate statistical significance at *p* < 0.001. The boxplots show the first and third quartile (box edges), median (middle line), and the data range (min/max = whiskers).

For individual traits, spore percent germination (SG) and time to germination (TG) were higher (*p* < 0.001) in homokaryons (mean SG = 81 ± 9.9%, mean TG = 11 days) than in dikaryons (mean SG = 55 ± 11.4%, mean TG = 13 days, Figure 4A and Supplementary Table 1). In contrast, dikaryotic strains had the highest hyphal growth (THL) and strongest exploration ability (HR) post-germination. Specifically, the total hyphal length (THL), the hyphal growth rate (HGS) and the HR were significantly higher (*p* < 0.001 for THL and HGS, *p* < 0.05 for HR) in dikaryotic strains (mean THL = 8115.8 μm, mean HGS = 195.6 μm/day, mean HR = 3383.9 μm) than for homokaryotic strains (mean THL = 3764.7 μm, mean HGS = 124.6 μm/day, mean HR = 2737.2 μm, Figures 4B–D). There were no links to nuclear status (homokaryons vs. dikaryons) for the remaining asymbiotic traits—*branching intensity* (*p* = 0.87), *number of germ tubes* (*p* = 0.15), *septae per hyphal unit* (*p* = 0.72), *spore area* (*p* = 0.21), and *spore diameter* (*p* = 0.067) (Supplementary Figure 5).

**Experiment 2**: *Symbiotic trait variation between homokaryotic and dikaryotic strains of R. irregularis.*

**FIGURE 5:**
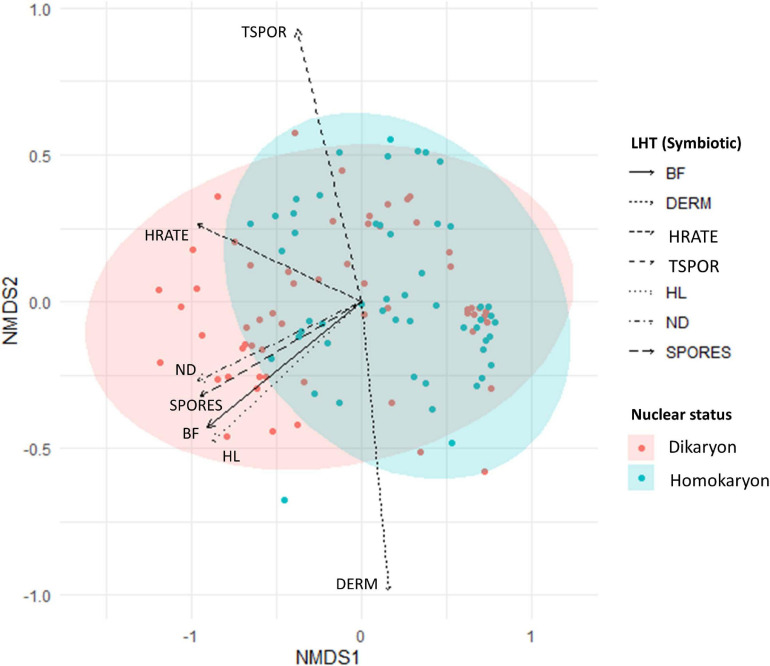
Non-metric multidimensional scaling (NMDS) of homokaryotic (blue dots) and dikaryotic (red dots) strains based on the measured life history traits during the symbiotic growth stage. Stress value = 0.08. The direction of the traits was obtained after fitting the measured traits in the ordination space. BF, branching factor; DERM, days until ERM emergence; HRATE, hyphal exploration rate; TSPOR, time to sporulation; THL, total hyphal length; ND, hyphal network density; SPORES, number of spores. Blue and red ellipse are 95% CI.

**FIGURE 6:**
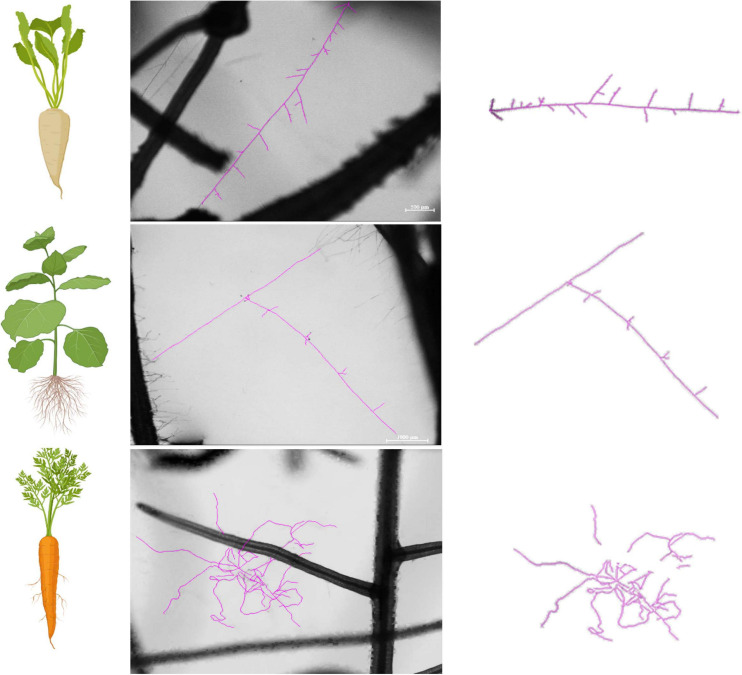
Hyphal growth patterns as affected by the host plant identity. We observed distinct hyphal growth patterns across strains, regardless of their genetic organization (Homokaryons vs. Dikaryons). Specifically, hyphae supported by *Cichorium intybus* resembled the morphology of a “fish bone” with regular, short-length branching patterns. Hyphae related to *Nicotiana benthamiana* was the least complex of the three and was mostly linear with limited, irregular, and noticeably short branches. Finally, hyphae supported by *Daucus carota* exhibited the most complex growth of the three with lengthy and abundant branches and a lot of three-dimensional growth. The observed differences are not due to different developmental stages since the growth patterns remain consistent throughout the hyphal network development.

When all symbiotic traits are considered, our analyses reveal significant differences between homokaryons and dikaryons (PERMANOVA, Pseudo-*F* = 18.35, *p* = 0.001) (Figure 5 and Supplementary Figure 6). We also find a significant host effect, where different hosts alter hyphal growth patterns of all strains investigated regardless of their nuclear status (PERMANOVA, Pseudo-*F* = 71.94, *p* = 0.001) (Figure 6). However, dikaryons grow significantly better than homokaryons when interacting with distinct hosts—i.e., dikaryons grew more with carrot and chicory, whereas homokaryons showed reduced growth with other hosts than carrot, as evident by the significant interaction between host plant and nuclear status (interaction—PERMANOVA, Pseudo-*F* = 4.13, *p* = 0.003).

**FIGURE 7:**
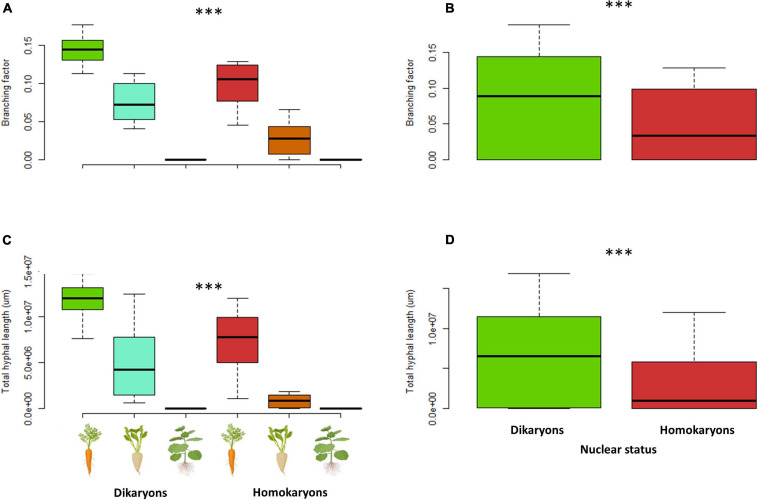
Branching factor **(A,B)** and total hyphal length **(C,D)** between four dikaryotic and four homokaryotic strains of *Rhizophagus irregularis* per root organ culture host used in the study. **(A,C)** Trait variation per nuclear status and host plant identity. **(B,D)** Trait variation per nuclear status. ^∗∗∗^indicates statistical significance at <0.001.

When each trait was examined separately, the *branching factor* is significantly different between dikaryotic and homokaryotic strains (*p* < 0.05) with dikaryotic strains producing more branched hyphae than homokaryotic strains. This trait was also significantly affected by the host identity (*p* < 0.001)—e.g., carrot ROC inducing a more complex hyphal network than chicory and tobacco ROCs; the latter surprisingly causing linear (unbranched) hyphal growth (Figure 7). Finally, dikaryons produced more complex hyphal networks across hosts compared to homokaryons (*p* < 0.01).

**FIGURE 8:**
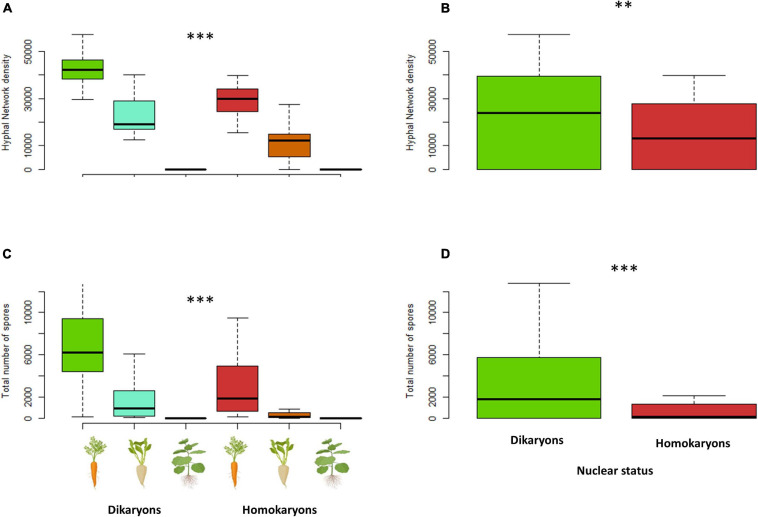
Hyphal network density **(A,B)** and total number of spores **(C,D)** variation between dikaryotic and homokaryotic strains of *Rhizophagus irregularis* per root organ culture host used in the study. **(A,C)** Trait variation per nuclear status and host plant identity. **(B,D)** Trait variation per nuclear status. ^∗∗^ indicates statistical significance at <0.01 and ^∗∗∗^ indicates statistical significance at <0.001.

The total *hyphal length* (HL) and *network density* were both significantly different (*p* < 0.001) between dikaryons (mean HL = 6.4 m) and homokaryons (mean HL = 3 m). The host plant identity had a significant effect on HL (*p* < 0.001) and ND (*p* < 0.05), with carrot ROC promoting higher hyphal growth (mean HL = 9.2 m) than chicory ROC (mean HL = 3.2 m) and tobacco ROC (mean HL = 0.0031 m). However, dikaryons always produced more hyphae (*p* < 0.05) and denser hyphal networks irrespective of the host plant identity (*p* < 0.01; Figures 7, 8).

On average, dikaryotic strains produced about 2.5 times more spores than homokaryotic strains (*p* < 0.001, mean = 3,097 vs. mean = 1,246 spores, respectively). *Spore number* was also significantly affected by the host plant identity (*p* < 0.001, Figure 8) with carrot ROC promoting higher spore production more (mean = 4,718) than chicory ROC (mean = 1,140) and tobacco ROC (mean = 1 spore). Dikaryons produced significantly more spores across hosts (interaction—*p* < 0.05) than homokaryons, which showed reduced spore production with both chicory and tobacco.

**FIGURE 9:**
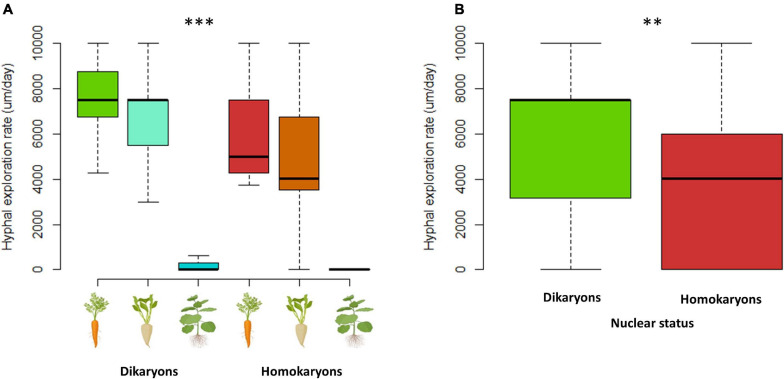
Hyphal exploration speed **(A,B)** variation between dikaryotic and homokaryotic strains of *Rhizophagus irregularis* per root organ culture host used in the study. **(A)** Trait variation per nuclear status and per ROC. **(B)** Trait variation per genetic. ** indicates statistical significance at <0.01 and *** indicates statistical significance at <0.001.

**FIGURE 10:**
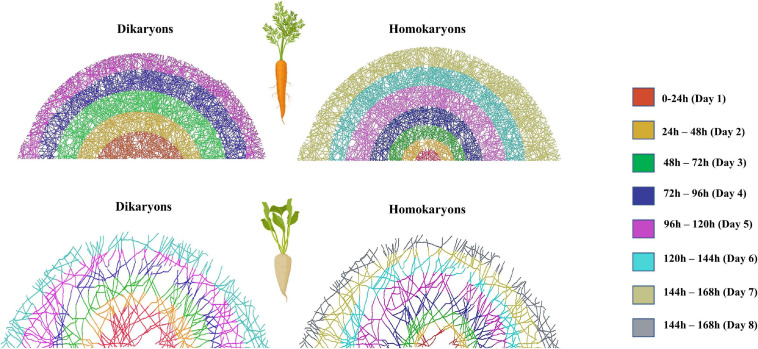
Exploration speed (time it takes for the hyphae to reach the edge of the hyphal compartment) variation between dikaryotic and homokaryotic strains of *Rhizophagus irregularis* when growing with **(A)** *Daucus carota* and **(B)** *Cichorium intybus* root organ culture. Different colors indicate different distance classes. Fewer color categories correspond with faster exploration speed. Dikaryotic strains exhibited faster exploration speed compared to homokaryotic across hosts. This image was created using Adobe Photoshop version 22.1.1. based on the hypha exploration speed (GSPEED). The schematic representation of the mycelium is an average representation based on the replicates per host plant species and nuclear status.

Mycelia of dikaryons were able to spread faster into the fungal compartment (*p* < 0.01), with an average production of hyphae of 5.7 vs. 3.7 mm/day for homokaryons. *Hyphal exploration rate* was also significantly affected by the host plant identity (*p* < 0.001) with tobacco ROC significantly slowing down both hyphal growth and exploration ability, irrespective of AMF strain (mean = 0.3 mm/day, compared to 7.2 mm/day for carrot ROC and 5.9 mm/day for chicory (Figures 9, 10). There was no significant correlation between hyphal growth and exploration ability (*p* = 0.1).

*Time to sporulation* did not differ between homokaryons and dikaryons (*p* = 0.58); however, this trait was heavily affected by the host plant identity (*p* < 0.001, Supplementary Figure 7). Regardless of nuclear status (*p* = 0.12), sporulation occurred sooner with tobacco ROC (mean GSPOR = 9.07) than with carrot ROC (mean GSPOR = 36.4) and chicory ROC (mean GSPOR = 32.4). *Days to ERM emergence* neither differed between dikaryotic and homokaryotic strains (*p* = 0.57), nor they were affected by the host plant identity (*p* = 0.46) (Supplementary Figure 7) or nuclear status. We did not observe a significant interaction between nuclear status and host plant identity (*p* = 0.88).

## Discussion

### *Rhizophagus irregularis* Nuclear Status Determines Life History Traits

The AMF intraspecific variability in phenotypic traits is known to exist both at the asymbiotic (Kokkoris et al., 2019a) and symbiotic stages (Hart and Reader, 2002; Koch et al., 2004, 2006; Munkvold et al., 2004; Ehinger et al., 2009). Here, we showed that some of this variability is linked to the nuclear status of *R. irregularis*, as we found contrasting fitness-related LHTs between homokaryons and dikaryons.

In the asymbiotic stage, homokaryotic spores germinate faster and more frequently compared to dikaryotic spores and thus may get a priority effect in symbiotic associations (Werner and Kiers, 2015). This could be especially important when plants are abundant, and roots are easily encountered. However, dikaryotic strains appear to grow significantly faster once their spores have germinated, producing a wider and more branched hyphal network, ensuring a more efficient exploration of the surrounding area. Therefore, the priority advantage of AMF homokaryons must rapidly decline as dikaryons eventually catch up in colonizing roots.

Dikaryons also outgrew homokaryons during the symbiotic stage. We found that dikaryons establish a larger hyphal network. A wider hyphal network implies a more efficient nutrient uptake for the host since the ERM acquires resources (Jakobsen et al., 1992). The ability to explore larger areas, and subsequently reach more nutrients, suggests that dikaryons could be more beneficial to plant partners than homokaryotic relatives—i.e., they should provide plants a larger access to nutrients. Future studies based on nutrient tracing should investigate whether hosts do actually obtain more nutrients from dikaryotic strains of *R. irregularis*, and whether they preferentially form symbioses with dikaryons rather than homokaryons when both strains coexist (and particularly when nutrients are scarce).

### What Could Be Responsible for Slower Germination and Faster Growth in *R. irregularis* Dikaryons?

The slower germination rates and faster post-germination growth and increased exploration capabilities of dikaryons are difficult to interpret. Perhaps, these result from initial difficulties in coordinating inter-nucleus genetic interactions in newly germinating spores. Alternatively, maintaining two nuclei instead of one may initially result in higher metabolic costs for dikaryotic spores (Kokkoris et al., 2021). However, once interactions are faster, owing to fine-tuning, hyphal growth could then proceed at full speed, thanks to complementary proteins produced by the coexisting parental genotypes. Although speculative, this hypothesis is supported by similar mechanisms seen in other heterokaryotic fungi (e.g., *Neurospora crassa* and *Agaricus bisporus*), where each nucleotype expresses unique proteins leading to increased fitness for the heterokaryotic strains (Gehrmann et al., 2018).

As dikaryons contain significantly more nuclei than homokaryons (Kokkoris et al., 2021), the presence of nucleophagy—i.e., targeted degradation of nuclei (Kokkoris et al., 2020a)—may also explain the faster growth rate of dikaryons. Indeed, in other fungi, this process is known to create a rapid flux of nutrients (phosphate and nitrate) when needed by the organism (presumably for growth) (Shoji et al., 2010), and it is thus possible that a similar mechanism may provide an advantage to dikaryons of AMF as well.

### Are AMF Dikaryons Pre-adapted for Host Plant Switching?

It was recently shown that AMF dikaryons can change the ratio of parental nuclei in response to hosts, possibly as a result of adaptive genetic interaction with the plant partner (Kokkoris et al., 2021). In support of this, we found that AMF dikaryons consistently produced more spores and more hyphae and a denser hyphal network irrespective of the host plant identity. A higher spore production rate was previously reported for strain A4 (Koch et al., 2004), and higher hyphal density was previously reported for strain C3 (Ehinger et al., 2009), both of which are now known to be dikaryons.

Hyphal density is key for establishment and functioning of the mycorrhizal symbiosis. Thus, our findings indicate that dikaryons may have an advantage over homokaryons when it comes to establishing mycorrhizal connections with different host plants, possibly as a result of their higher genetic diversity (two nucleotypes) and their ability to regulate these nuclear states in response to their plant host (Kokkoris et al., 2021). Within this context, the ability of AMF dikaryons to interact more efficiently across a wider host range suggests dikaryons are being more generalist than homokaryons and may thus be encountered in ecosystems that harbor more diverse plant communities.

### Ecological and Economic Implications of AMF Nuclear Status

The ERM of a single AMF strain can interconnect with numerous distinct plants and form a common mycorrhizal network (CMN) (Giovannetti et al., 2004). CMNs enable resource redistribution among coexisting plants, resulting in higher plant survival, fitness and diversity (Hart et al., 2003), and community composition (Hart et al., 2001; Zobel and Öpik, 2014). The present study suggests that AMF nuclear status may be relevant for hyphal network establishment. Future studies should now examine this critical question by investigating the potential beneficial effects of AMF dikaryons for plant communities, and determine their distribution and frequency in the field and across distinct environmental conditions. Accounting for AMF nuclear status in ecological models could be key for answering questions of mycorrhizal ecology (Kokkoris et al., 2020b).

Our findings also have implications for the fungal bio-inoculum industry. The most commonly used strain for inoculants (*R. irregularis* DAOM197198—homokaryon) grows fast in carrot ROC and produces large quantities of spores (Douds, 2002; Rosikiewicz et al., 2017). However, there were cases in which it reduced host nutritional benefits (Kokkoris and Hart, 2019a, b; Kokkoris et al., 2019a) and failed to establish in large-scale inoculation trials (Farmer et al., 2007; Kokkoris et al., 2019b; Thomsen et al., 2021). In this study, DAOM197198 grew well with carrot as host plant and produced the most spores among all homokaryons, but surprisingly ranked last in terms of growth and spore production with the other two host plants, indicating host preference. In contrast, the observed ability of the *R. irregularis* dikaryons to grow better across a wider host range, a trait that could also translate into higher benefits to host plants, suggests that AMF dikaryons are more reliable inocula.

Besides DAOM197198, to our knowledge, all the *R. irregularis* strains used in inoculants, and the majority of the *R. irregularis* strains growing in AMF collections (with the exception of the five known dikaryotic *R. irregularis* strains), are homokaryotic (Kokkoris et al., 2021). With single spore *in vitro* inoculations being a necessary step for the establishment of a pure strain culture, it is possible that the observed dominance of homokaryons over dikaryons *in vitro* is due to the increased germination ability of homokaryons.

## Conclusion

In conclusion, the present work shows that AMF homokaryons differ from AMF dikaryons in asymbiotic and symbiotic LHTs. Our study provides evidence that the LHTs of the strains can be predicted by their nuclear status, regardless of their phylogenetic divergence (Savary et al., 2018) and despite the high intraspecific variation that exist in *R. irregularis* (Koch et al., 2004). Because the traits that we examined affect fungal and plant performance, this work has immediate implications for understanding the links between AMF nuclear status and mycorrhizal ecology. As such, these findings lay a foundation for future work that examines functional differences between AMF homokaryons and dikaryons *in plantae* and within AMF populations, including gene expression (Robbins et al., 2021). Lastly, the findings of this study have direct implications in efforts to produce efficient mycorrhizal fungal inocula.

## Data Availability Statement

The raw data supporting the conclusions of this article can be found at the Supplementary Material.

## Author Contributions

VK and NC designed the study and wrote the manuscript. ES, VK, and CC performed the experiments. All authors reviewed the manuscript.

## Conflict of Interest

The authors declare that the research was conducted in the absence of any commercial or financial relationships that could be construed as a potential conflict of interest.

## Publisher’s Note

All claims expressed in this article are solely those of the authors and do not necessarily represent those of their affiliated organizations, or those of the publisher, the editors and the reviewers. Any product that may be evaluated in this article, or claim that may be made by its manufacturer, is not guaranteed or endorsed by the publisher.
