# Supplementary material for: Homo- and Dikaryons of the Arbuscular Mycorrhizal Fungus Rhizophagus irregularis Differ in Life History Strategy
Source: Front Plant Sci. 2021 Aug 5;12:715377. doi: 10.3389/fpls.2021.715377 (PMC8374082; doi:10.3389/fpls.2021.715377)
Supplement: Supplementary file 1 [file Data_Sheet_1.pdf]

Table S1: Nuclear status, MAT-loci, H+-ATPase group, and percent spore germination (after evaluating the germination of 40 spores per strain in experiment 1) of the ten studied isolates of *Rhizophagus irregularis*. The column (# spore used) includes the standardized spore number to initiate colonization for experiment #2 in order to ensure at least 10 viable spores per plate.

**Strain**	**DAOM**	**Nuclear status**	**MAT-type**	**Group** (H+-ATPase)	**Germination (%)**	**# spores used for EXP # 2**
A4	664343	Dikaryon	MAT1 & MAT2	Group#3	55	18
A5	664344	Dikaryon	MAT3 & MAT6	Group#4	55	18
SL1	240409	Dikaryon	MAT1 & MAT5	Group#4	41	25
G1	970895	Dikaryon	MAT1 & MAT5	Group#1 or 2*	69	14
330	229455	Homokaryon	MAT2	Group#1 or 2*	81	12
197198	197198	Homokaryon	MAT4	Group#4	92	11
101	240448	Homokaryon	MAT5	Group#4	85	12
66	240720	Homokaryon	MAT3	Group#3	82	12
98	240446	Homokaryon	MAT6	Group#4	65	14
Cuba8	984909	Homokaryon	MAT1	Group#1 or 2*	0	NA

*The strains G1, 330 and Cuba8 belong to the same clade. Since these strains were not examined by Savary et al., 2018 it is unknown whether they belong to the group "1" or "2" based on their classification but they are distinct from groups "3" and "4". The distinct groups the strains belong to based on previous published phylogenies (Kokkoris et al., 2021; Ropars et al., 2016; Savary et al., 2018).

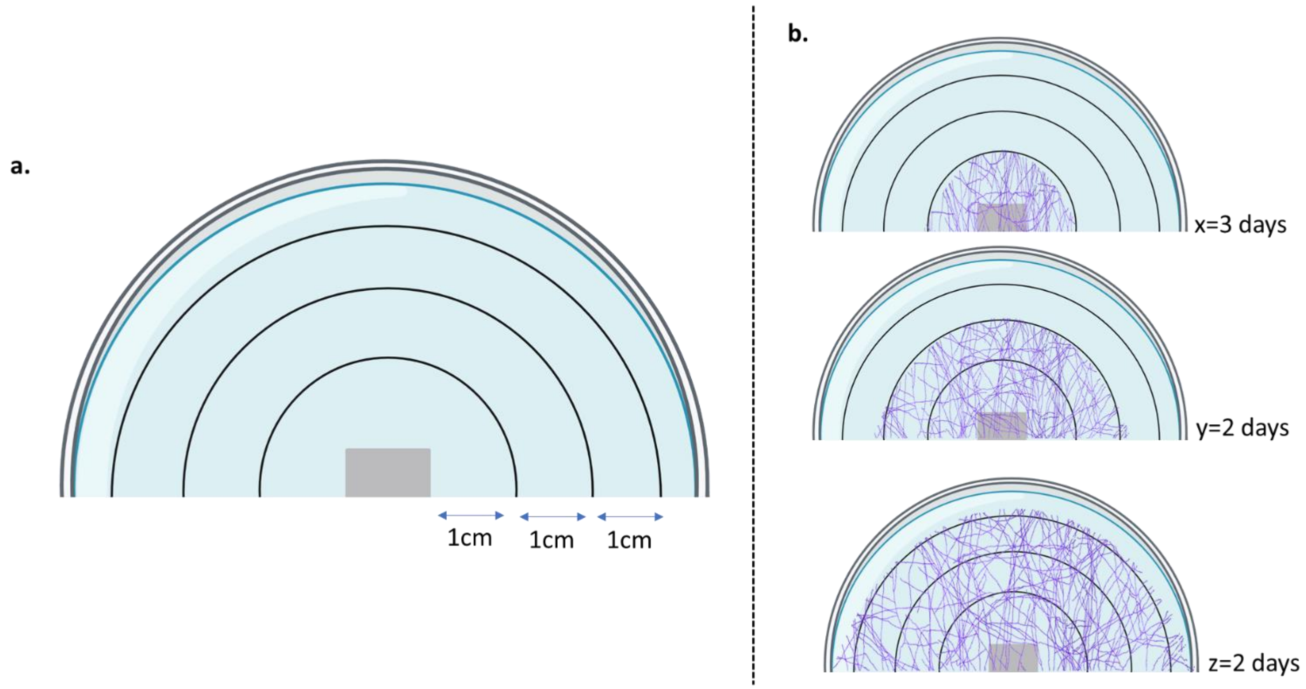

Figure S1. Hyphal exploration rate (HRATE). In each Petri dish, semicircles were drawn (1 cm intervals). Hyphae was monitored daily for growth and the day it took for the hypha to travel 1 cm into the fungal compartment from the moment the first hypha crossed the bridge (in days). **x** is the time it took for hyphae to reach the first semi-circle with the bridge as starting point. **y** is the time it took for the hyphae to reach the second semi-circle with the first semi-circle as a starting point. z is the time it took for the hyphae to reach the third semi-circle with the second semi-circle as a starting point. The average hyphal exploration speed was calculated in days using this formula: GSPEED = (x+y+z)/3.

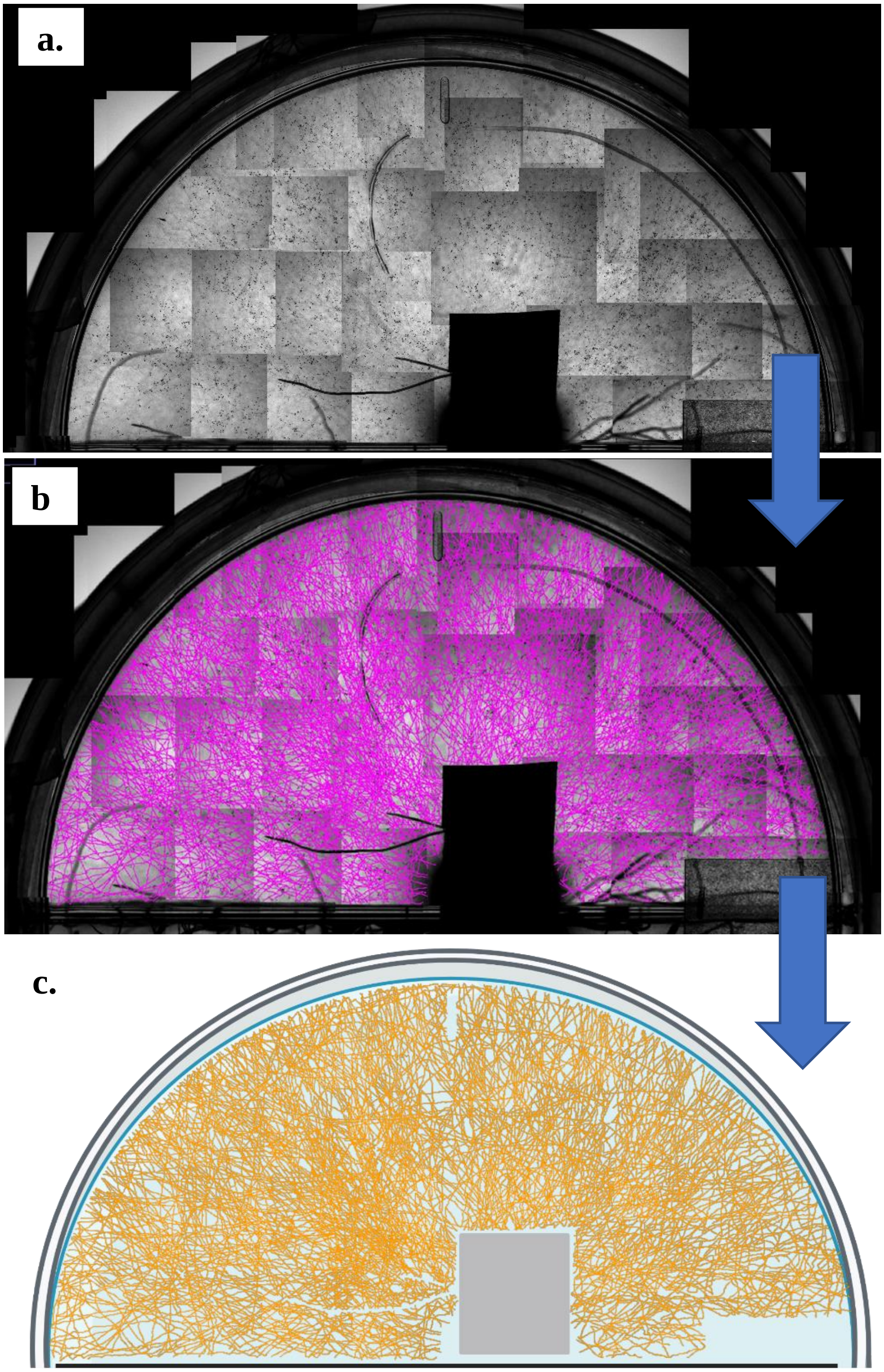

Figure S2. Hyphal trait quantification during the symbiotic stage. a) The entire hyphal compartment of a Petri dish was analyzed after stitching multiple images produced via microscopy together. b) The hyphal network was tracked semi-automatically using the Neuron J Plugin in FIJI (ImageJ v. 1.53c) (Schindelin et al., 2012) and c) the traced network was exported without background (.png with transparent background) using Adobe Photoshop version 22.1.1. and the final figure was created using BioRender (https://biorender.com). To correct for size variation of the filter bridge between plates, an area was removed from each image prior to determination of the hyphal traits.

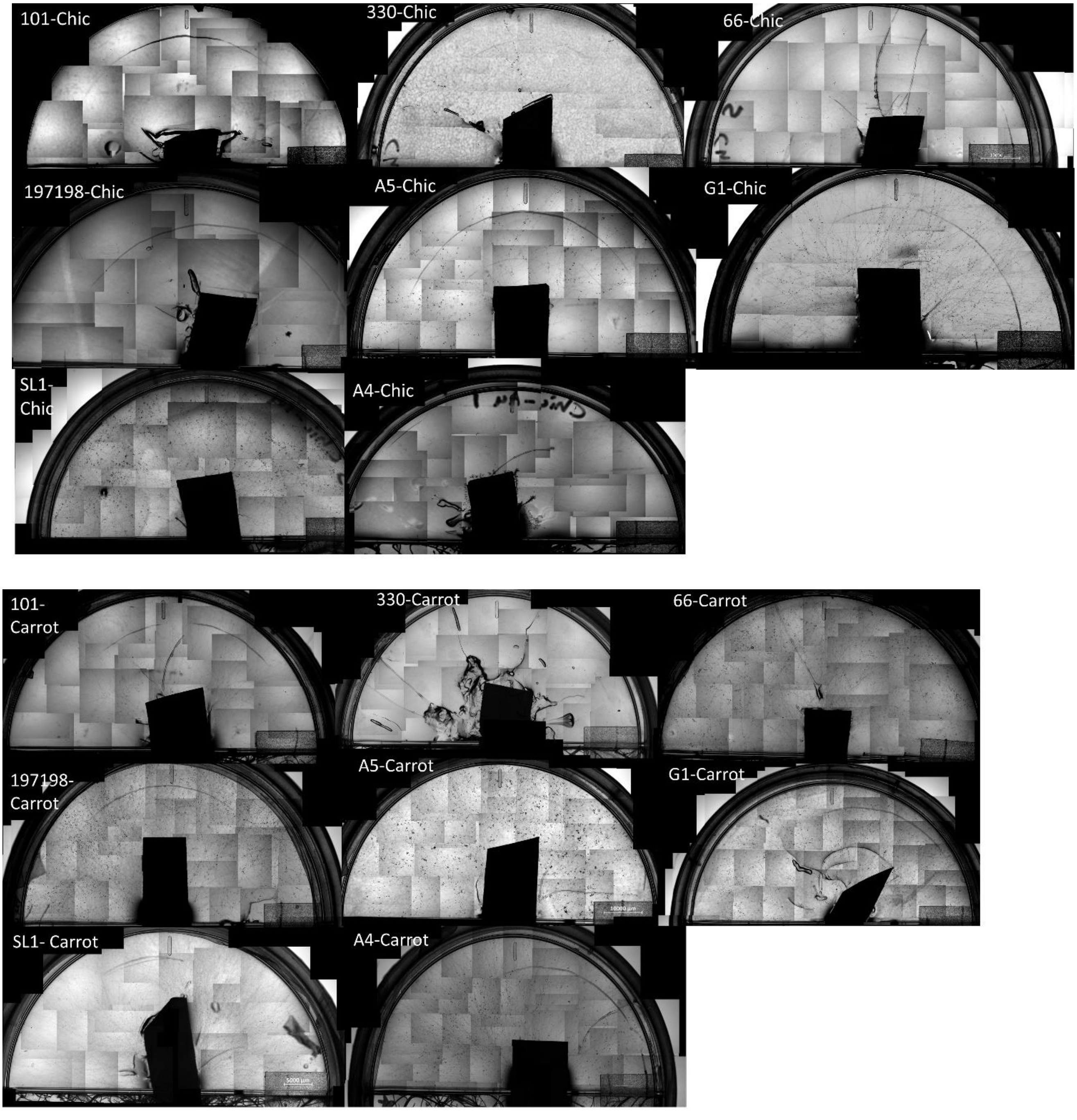

Figure S3. Additional representative examples of the growth variation between homokaryotic and dikaryotic *R. irregularis* strains when growing with chicory and carrot root organ cultures. We used a microscope (Zeiss AxioZoom.V16) and the function "panorama" in the software ZEN 2.3 software (Carl Zeiss MicroImaging, Gottingen, Germany) to acquire complete stitched images of the entire fungal compartment of each petri plate.

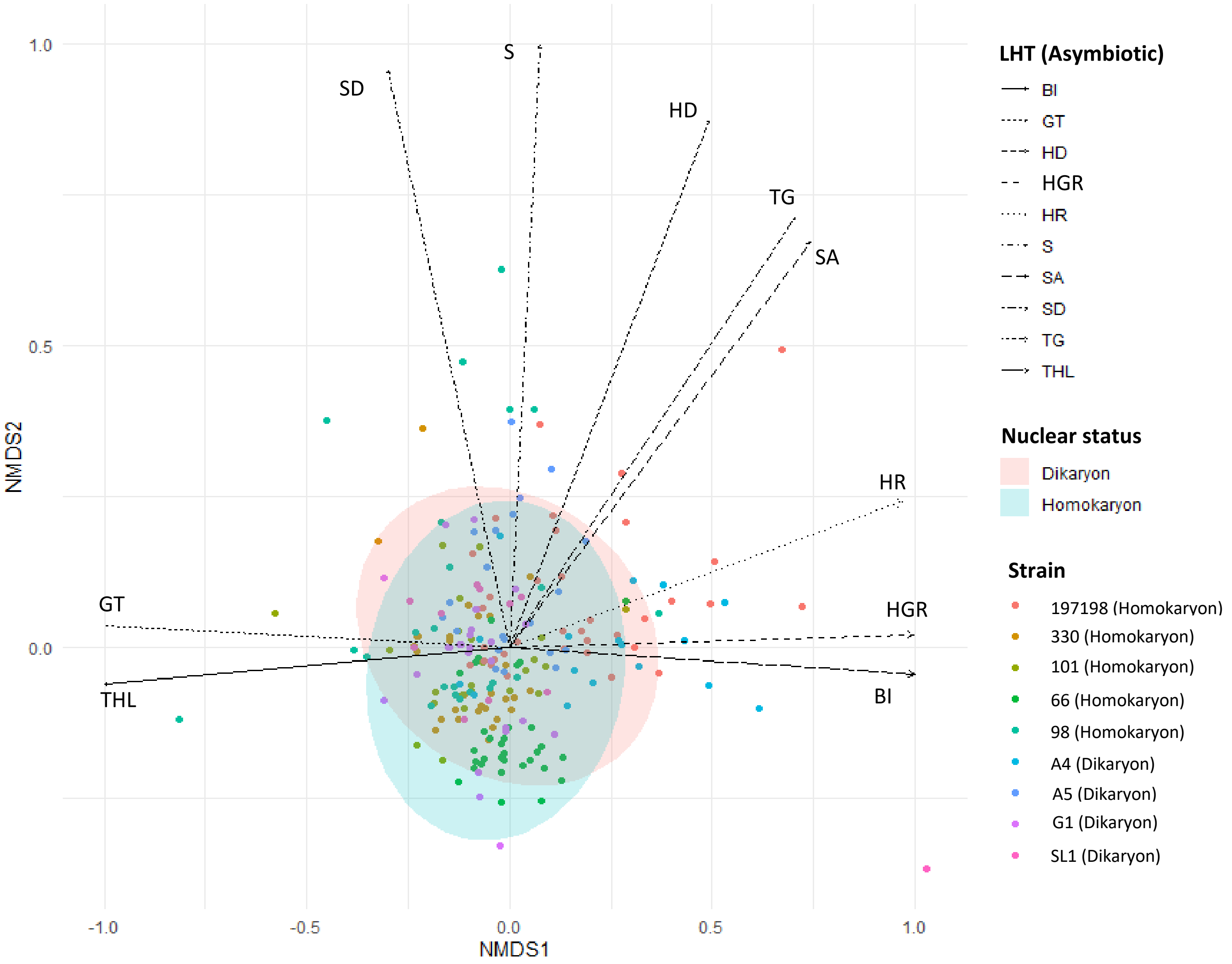

Figure S4. Non-metric multidimensional scaling (NMDS) of five homokaryotic and four dikaryotic strains of *Rhizophagus irregularis* (different colour per strain) based on the life history traits during the **asymbiotic** growth stage. Stress value = 0.1. The direction of the traits were obtained after fitting each measured trait in the ordination space. SD=Spore diameter, SA=Spore area, GT=Number of germ tubes, HR=Hyphal reach, HD=Hyphal diameter, TG=Time to germination, THL=total hyphal length, BI=branching intensity, S=Septa per hyphal unit, HGR=Hyphal growth rate. Blue and red ellipse are 95% confidence interval per nuclear status (Homokaryons vs Dikaryons).

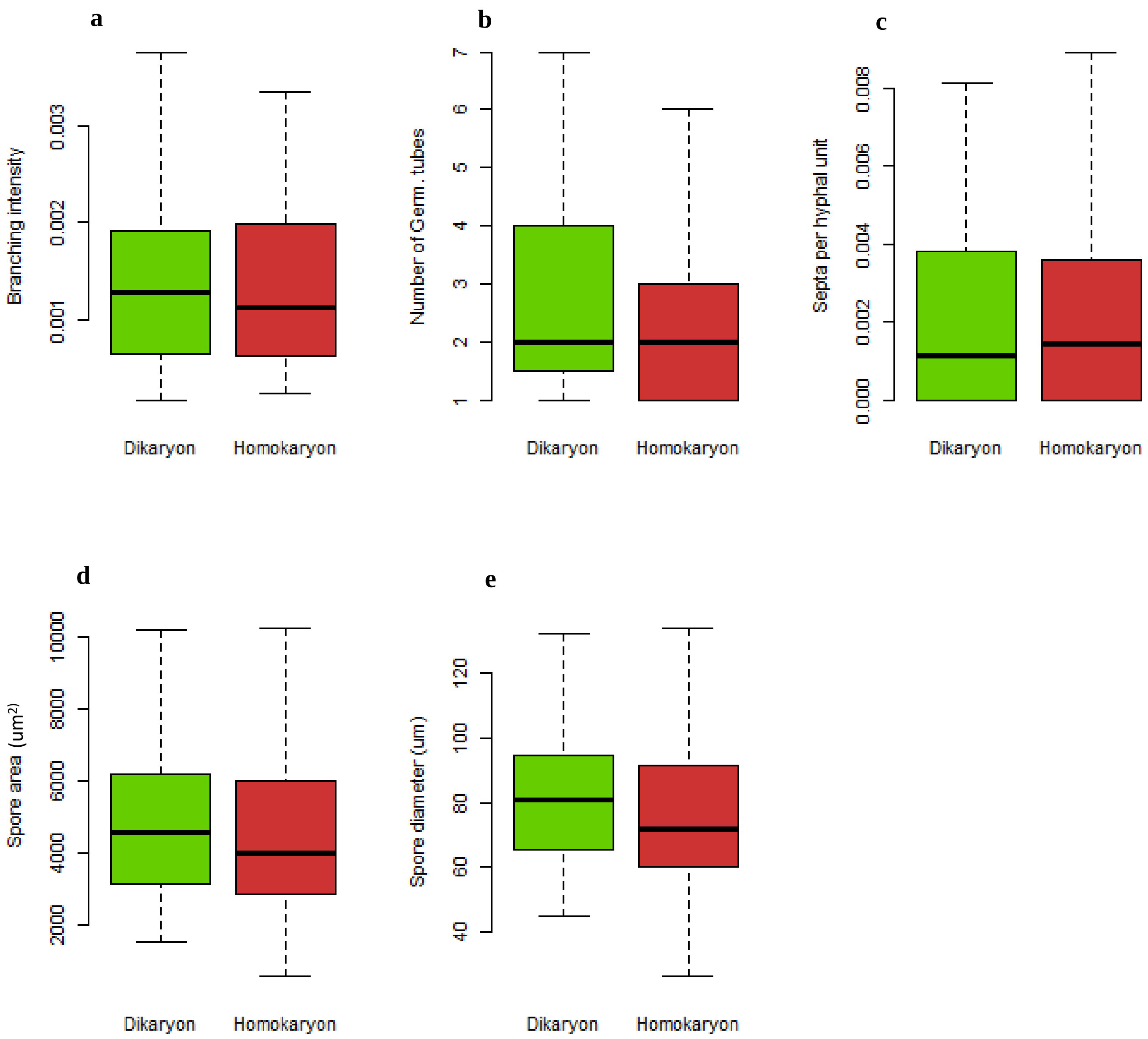

Figure S5. Trait variation between four dikaryotic (green boxplots) and five homokaryotic (red boxplots) strains of *Rhizophagus irregularis* during the asymbiotic growth stage**. a)** Branching intensity, **b)** Number of germination tubes, **c)** number of septae per hyphal unit **d)** spore area and **e)** spore diameter. Statistical significance was evaluated using the nonparametric Kruskal–Wallis test, but none was found for these traits. The boxplots show the first and third quartile (box edges), median (middle line), and the data range (min/max = whiskers).

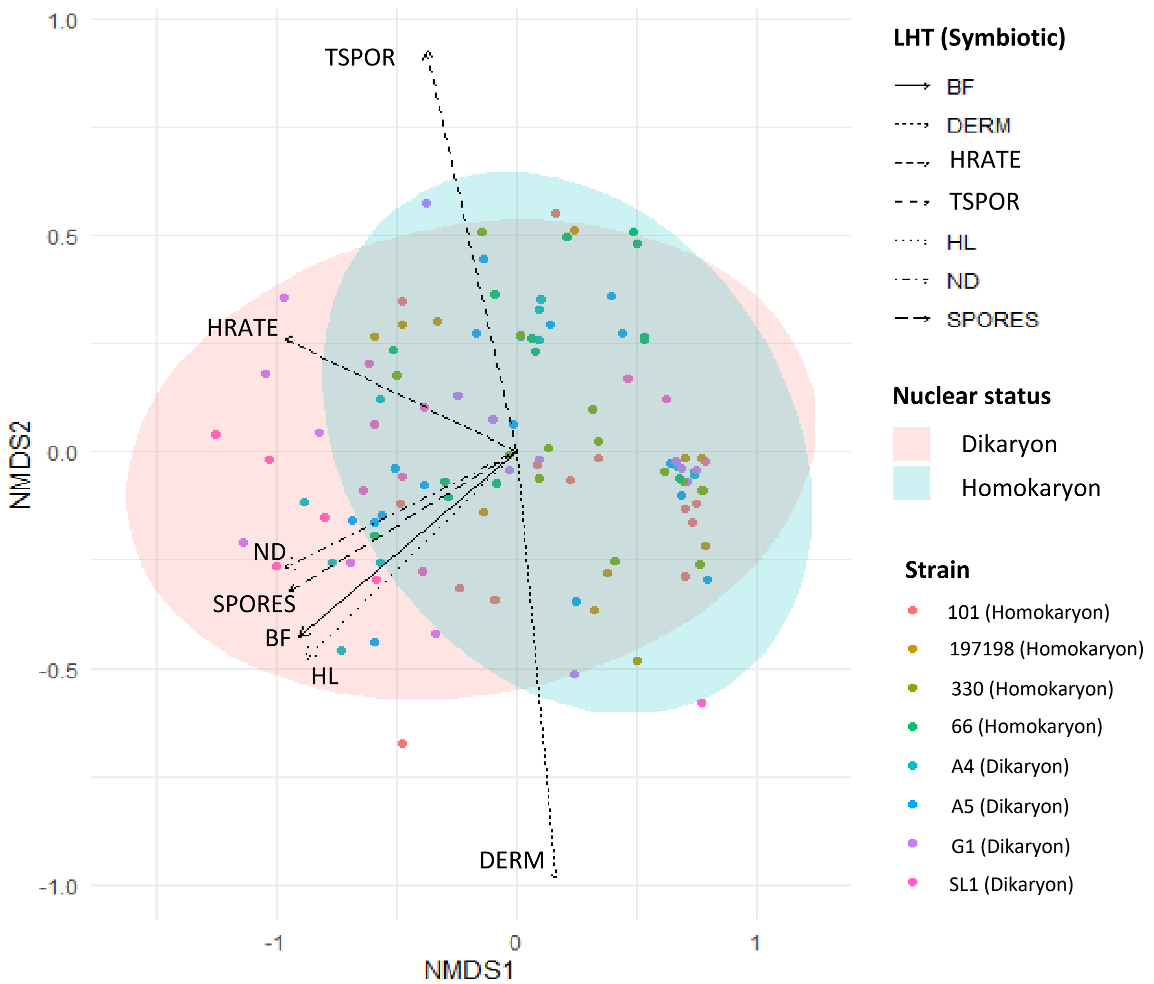

Figure S6. Non-metric multidimensional scaling (NMDS) of four homokaryotic and four dikaryotic strains of *Rhizophagus irregularis* (different colour per strain) based on the life history traits during the **symbiotic** growth stage. Stress value = 0.08. The direction of the traits were obtained after fitting each measured trait in the ordination space. BF = Branching factor, DERM = Days Until Extraradical Mycelium Emergence, HRATE =Hyphal exploration rate, TSPOR = Time to Sporulation, HL = Total Hyphal Length, ND = Hyphal Network density, SPORES = Number of spores. Blue and red ellipse are 95% confidence interval per nuclear status (Homokaryons vs Dikaryons).

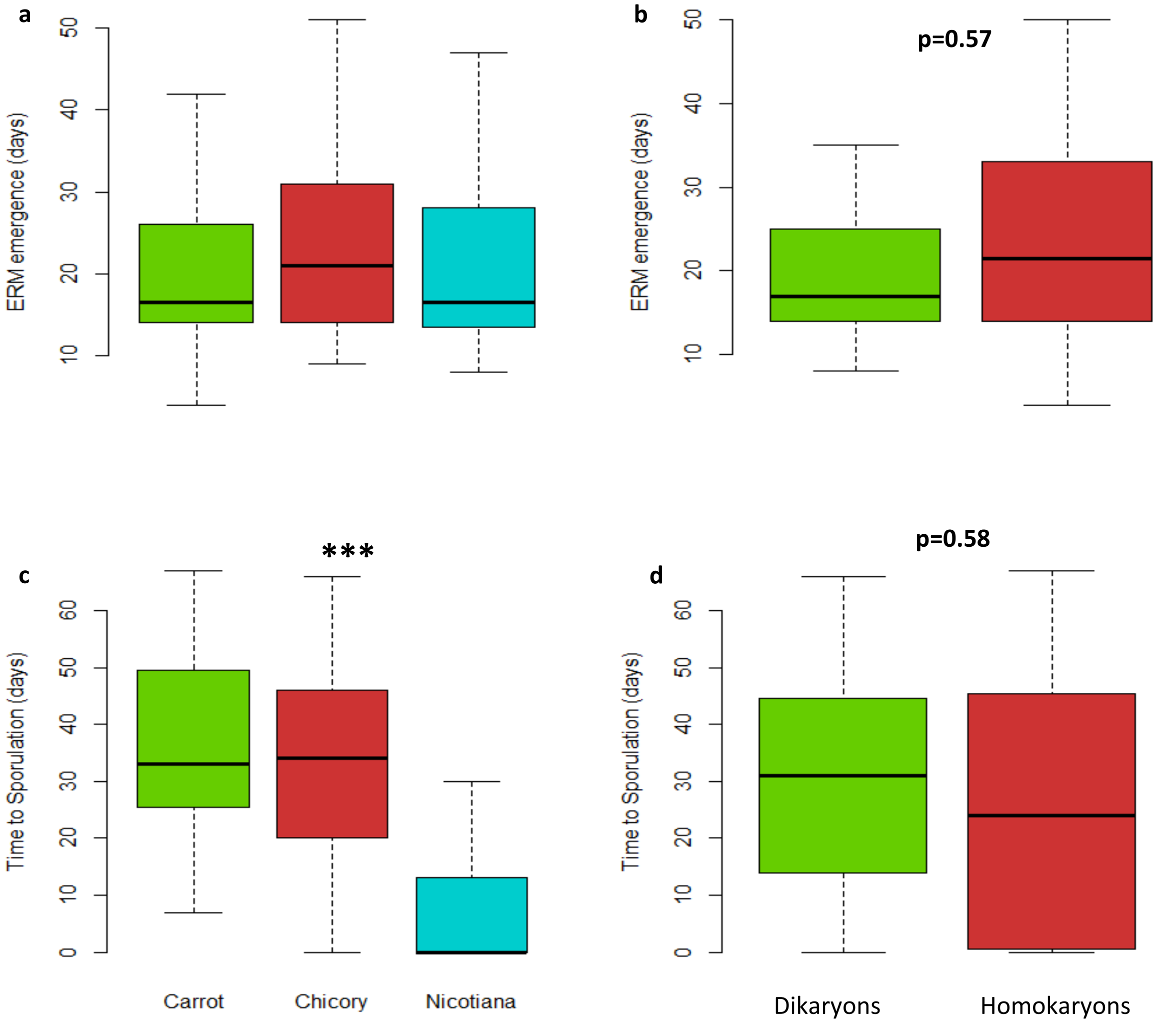

Figure S7. Extraradical Mycelium Emergence **(a, b)** and Time to sporulation **(c, d)** variation between dikaryotic and homokaryotic strains of *Rhizophagus irregularis* per root organ culture host plant species used in the study**. a & c)** Trait variation per nuclear status and per root organ culture. **b & d).** Trait variation per nuclear status. DERM is the days required for the extraradical mycelium to emerge and GSPOR is the time it takes the strain to form spores. *** indicate statistical significance at <0.001.

Supplemental references

Kokkoris, V., Chagnon, P.-L., Yildirir, G., Clarke, K., Goh, D., MacLean, A. M., et al. (2021). Host identity influences nuclear dynamics in arbuscular mycorrhizal fungi. Curr. Biol. doi:10.1016/j.cub.2021.01.035.

Ropars, J., Toro, K. S., Noel, J., Pelin, A., Charron, P., Farinelli, L., et al. (2016). Evidence for the sexual origin of heterokaryosis in arbuscular mycorrhizal fungi. Nat. Microbiol. 1. doi:10.1038/nmicrobiol.2016.33.

Savary, R., Masclaux, F. G., Wyss, T., Droh, G., Cruz Corella, J., Machado, A. P., et al. (2018). A population genomics approach shows widespread geographical distribution of cryptic genomic forms of the symbiotic fungus *Rhizophagus irregularis*. ISME J. 12, 17–30. doi:10.1038/ismej.2017.153.
